# The Maximal Lactate Steady State Workload Determines Individual Swimming Performance

**DOI:** 10.3389/fphys.2021.668123

**Published:** 2021-04-26

**Authors:** Gernot O. Hering, Jens Stepan

**Affiliations:** ^1^Department of Sport and Health Science, University of Konstanz, Konstanz, Germany; ^2^Department of Obstetrics and Gynaecology, Paracelsus Medical University, Salzburg, Austria

**Keywords:** lactate threshold, maximal lactate steady state, swimming, performance testing, exercise physiology

## Abstract

The lactate threshold (LT) and the strongly related maximal lactate steady state workload (MLSS_W_) are critical for physical endurance capacity and therefore of major interest in numerous sports. However, their relevance to individual swimming performance is not well understood. We used a custom-made visual light pacer for real-time speed modulation during front crawl to determine the LT and MLSS_W_ in a single-exercise test. When approaching the LT, we found that minute variations in swimming speed had considerable effects on blood lactate concentration ([La^−^]). The LT was characterized by a sudden increase in [La^−^], while the MLSS_W_ occurred after a subsequent workload reduction, as indicated by a rapid cessation of blood lactate accumulation. Determination of the MLSS_W_ by this so-called “individual lactate threshold” (ILT)-test was highly reproducible and valid in a constant speed test. Mean swimming speed in 800 and 1,500 m competition (S-Comp) was 3.4% above MLSS_W_ level and S-Comp, and the difference between S-Comp and the MLSS_W_ (Δ S-Comp/MLSS_W_) were higher for long-distance swimmers (800–1,500 m) than for short- and middle-distance swimmers (50–400 m). Moreover, Δ S-Comp/MLSS_W_ varied significantly between subjects and had a strong influence on overall swimming performance. Our results demonstrate that the MLSS_W_ determines individual swimming performance, reflects endurance capacity in the sub- to supra-threshold range, and is therefore appropriate to adjust training intensity in moderate to severe domains of exercise.

## Introduction

Changes in physical endurance capacity require precise adaptation of various physiological processes. The exact knowledge of the exercise associated stimulus/response pattern, and the availability of reliable and valid diagnostic systems are prerequisites to induce particular homeostatic perturbations and to implement successful training interventions (Fluck and Hoppeler, 2003). Frequency, duration and volume are the major parameters for controlling training load. However, selective adjustment of training intensity is the most potent trigger of endurance capacity specification (Bishop et al., 2019; Jamnick et al., 2020).

At some point, a steadily increasing workload (WL) will lead to rapid arterial lactate accumulation. This so-called lactate threshold (LT; Brooks, 1985a) is closely related to the maximal lactate steady state workload (MLSS_W_), the highest WL with equilibrium between lactate production and removal and thus constant arterial lactate concentration [La^−^] (Donovan and Brooks, 1983; Brooks, 1985a,b; Heck et al., 1985; Stanley et al., 1985). For the following reasons, others and we suggest the MLSS_W_ as a major submaximal parameter for individual training intensity recommendations (Billat et al., 2003; Faude et al., 2009; Hering et al., 2018; Jamnick et al., 2020): First, the MLSS_W_ is highly reproducible, valid, and objective across endurance sports (Hering et al., 2018; Jamnick et al., 2020). Second, it has been repeatedly shown that exercise in the MLSS_W_ range induces key neuromuscular and metabolic adaptations (Bergman et al., 1999; Dubouchaud et al., 2000; Messonnier et al., 2013; Figueiredo et al., 2014; de Araujo et al., 2015; Hering et al., 2018).

At the MLSS_W_, minute WL increments cause a rapid increase in [La^−^], which is very likely due to an imbalance between lactate production and removal within the complex shuttle network of lactate metabolism (Donovan and Brooks, 1983; Brooks, 1985b; Stainsby and Brooks, 1990; Messonnier et al., 2013). An underlying neuromuscular mechanism is increased activity in motor units with high lactate efflux. If activity in lactate-consuming motor units remains unchanged, this triggers rapid arterial lactate accumulation in relation to power output. In addition, changes in the intra- and inter-muscular innervation, muscle-fiber recruitment pattern, fatigue, and in molecular signaling cascades contribute to perturbations of lactate homeostasis at the MLSS_W_ (Hering et al., 2018).

The traditional method of MLSS_W_ determination requires several tests on different days and is therefore time-consuming and inappropriate for daily use (Heck et al., 1985; Figueiredo et al., 2014; Pelarigo et al., 2017; Jamnick et al., 2020). To circumvent these problems, we recently introduced a single-exercise test in running and cycling (Hering et al., 2018). Here, we have adapted the test algorithm for swimming and can reliably determine the MLSS_W_ in less than 1 h. Our data not only recapitulate a remarkably fine-tuned regulation of lactate metabolism at the MLSS_W_ in swimming, but also provide evidence that the MLSS_W_ reflects physical endurance capacity in ranges above the MLSS_W_. Consequently, we suggest the MLSS_W_ as an important submaximal parameter for the precise adjustment of training stimuli in moderate to severe domains of exercise.

## Materials and Methods

### Subjects

Fifty-five single-exercise swimming tests [50 amateur athletes with regular participation in competitions up to national championships aged 11–32 years (19 ± 0.74 years, 27 females)] were selected for ILT and MLSS_W_ determination. Each participant gave written, informed consent after being provided a detailed description of the study requirements and protocols. The experimental procedures were approved by the University of Konstanz Institutional Review Board and were performed in accordance with the ethical standards of the Government of Baden Württemberg. All subjects were healthy and avoided heavy exercise and maintained a normal diet at least 2 days before testing.

### Pacing System

**Figure 1:**
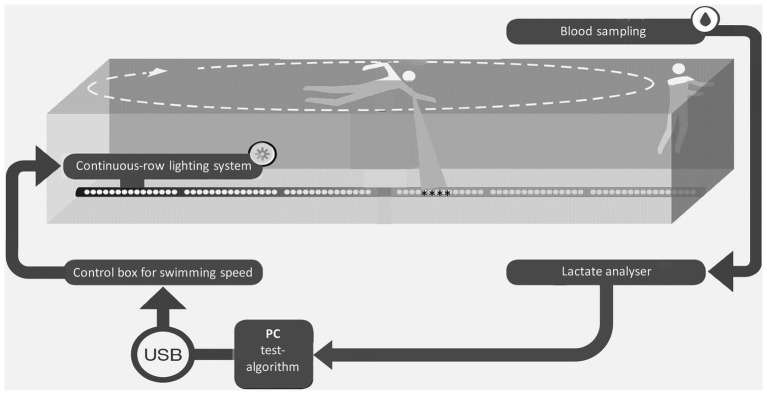
Experimental arrangement of the individual lactate threshold-test in swimming.

The visual light pacing system consisted of 25 interconnected polycarbonate tubes, each with four light-emitting diodes. The 100 red lights could be switched on and off individually *via* a microcontroller connected to a PC (control box for swimming speed, Figure 1). A waterproof power cable and a steel cable ensured a constant distance of 25 mm between the diodes (Figure 1). The software-controlled, portable pacing system was located on the pool floor and the swimmer followed the continuously moving light strip (Figure 1).

### Study Protocol of Swim Tests

**Figure 2:**
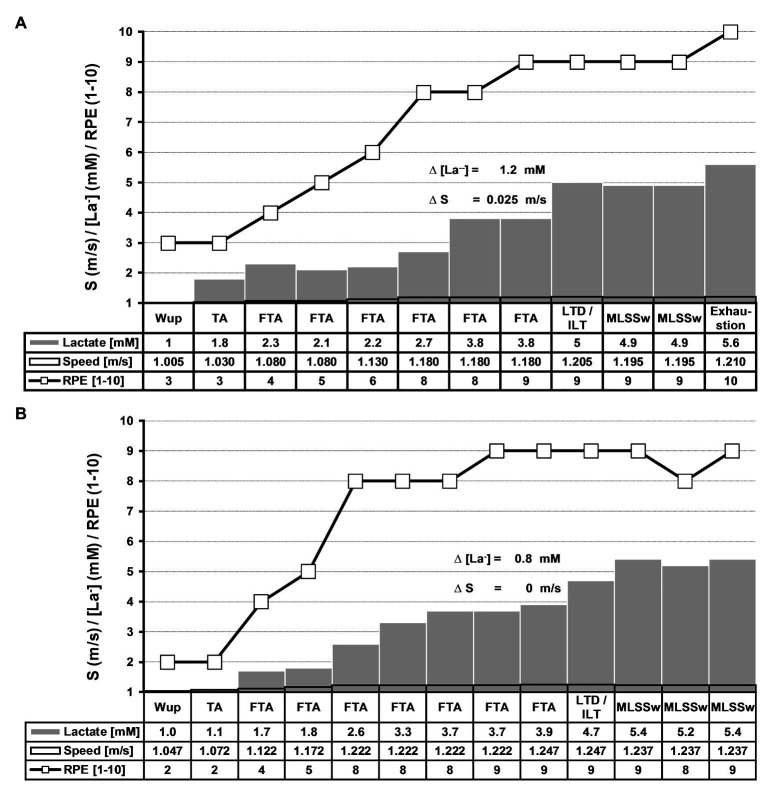
Experimental protocols of two typical ILT-tests. **(A)** LT detection with threshold criterion 1 (TC1) and **(B)** with threshold criterion 2 (TC2; Figure 3). The basic experimental protocol is identical on the treadmill, bicycle ergometer, and for swimming (for detailed information, see Figures 4A,B; Hering et al., 2018). After warm-up (W_UP_), threshold adjustment (TA), and fine threshold adjustment (FTA; see section “Material and Methods”), a step-like blood lactate accumulation occurred at the individual lactate threshold (ILT; ∆[La^−^]) after 1 (TC1; **A**) or after up to six steps (TC2; **B**). Immediately or one increment after a comparable slight workload reduction (0.01–0.015 m/s), the lactate accumulation slowed down, indicating the maximal lactate steady state workload (MLSS_W_). Real-time workload adjustments were set in accordance to blood lactate level in the previous exercise period, explaining the variable test durations even intraindividually (data not shown). [La^−^], blood lactate concentration; RPE, rate of perceived exertion; S, speed.

**Figure 3:**
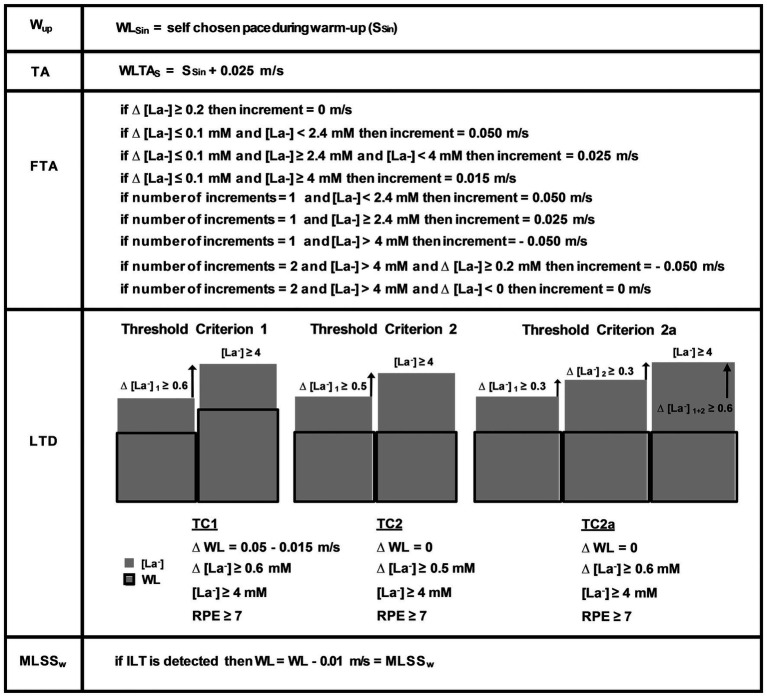
ILT-test algorithms. Swimming pace (S_Sin_) during warm-up (W_UP_) was self-chosen. The fine threshold adjustment (FTA) was executed in accordance to test duration and blood lactate dynamics. The current threshold criteria (TC) 1, 2 and 2a for determination of the individual lactate threshold (ILT), have been developed into their current form by continuously incorporating ILT-test data. After determination of the individual lactate threshold (ILT), the lactate accumulation usually slowed down after a slight workload reduction indicating the MLSS_W_. [La^−^], blood lactate concentration; MLSS_W_, maximal lactate steady state workload; RPE, rate of perceived exertion; and WLTAs, initial workload adaptation.

**Figure 4:**
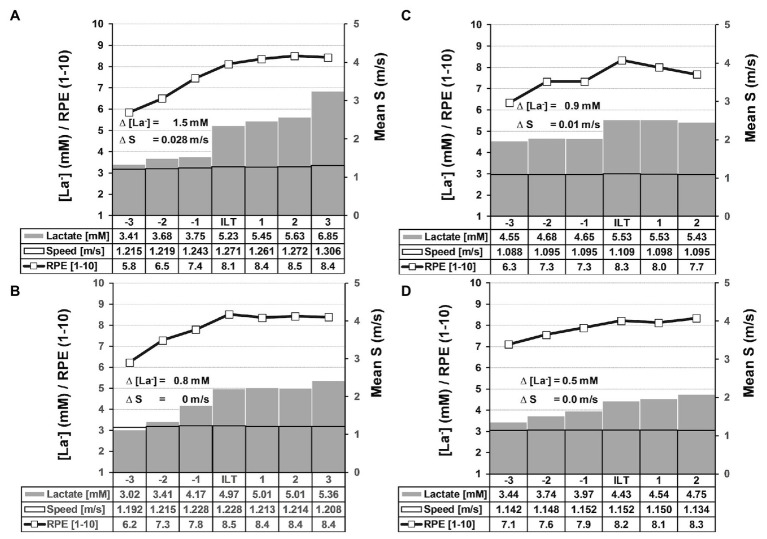
Summary of (individual lactate threshold) ILT-tests and constant speed (CS)-tests. **(A,B)** Summary of ILT-tests that met threshold criterion 1 (**A**, *n* = 18) or 2 (**B**, *n* = 37). Group mean values are shown for blood lactate concentration (mM), speed (m/s), and RPE (1–10). Note that the step-like lactate accumulation at the ILT in response to a minute increase in swimming speed. **(A)** Threshold criterion 1: ∆[La^−^] = 1.5 ± 0.237 mM, ∆S = 0.028 ± 0.003 m/s. **(B)** Threshold criterion 2: ∆[La^−^] = 0.8 ± 0.065 mM. **(C/D)** Summary of (CS)-tests that met threshold criterion 1 (**C**, n = 4) or 2 (**D**, n = 9). Group mean values are shown for blood lactate concentration (mM), Speed (m/s), and RPE (1–10). Note that the step-like lactate accumulation at the ILT in response to a minute increase in swimming speed. **(C)** Threshold criterion 1: ∆[La^−^] = 0.9 ± 0.17 mM, ∆S = 0.01 m/s, *n* = 4. **(D)** Threshold criterion 2: ∆[La^−^] = 0.5 ± 0.05 mM. Data are mean ± SE. [La^−^], arterial lactate concentration; RPE, rate of perceived exertion; S, speed.

The basic protocol for the so called individual lactate threshold (ILT)-test was identical to previous treadmill and bicycle ergometer tests (Hering et al., 2018; for detailed explanation see below, and Figures 2A,B, 3). The ILT-test consists of five distinct stages: (i) warm-up (W_UP_), (ii) threshold adaptation (TA), (iii) fine threshold adaptation (FTA), (iv) lactate threshold detection (LTD) by means of two threshold criteria (TC), followed by (v) a minimal workload reduction for verification of the MLSS_W_ (Figures 2A,B, 3, 4A,B). It is important to note that the ILT-test does not have a fixed number of stages and the swimming speed is set in relation to the [La^−^] from the previous step.

For the present study, all 55 datasets were retrospectively analyzed with the latest TC. The TC were refined over several years to optimize the test procedure (e.g., faster FTA), the results however, have not changed. Each subject performed a warm-up in a 25 m swimming pool at a self-chosen pace (S_Sin_), followed by a 60-s period to calibrate the visual light pacing system. The initial swimming speed was set 0.025 m/s above S_Sin_. The fine-tuning toward the LT was achieved by means of several steps with slight WL increments until a TC was realized (Figures 2A,B, 3). Every WL increment/reduction was set in accordance to lactate concentration from the previous step (Figures 2A,B, 3). We favored a 3–4-min step duration to ensure a metabolic and blood circulation adaptation phase of at least 3 min after a potential WL variation (Weltman et al., 1990; Bentley et al., 2007). The blood sample was taken when the swimmer reached the end of the pool after a step duration of at least 180 s. [La^−^] was measured [Arkray Lactate Pro® 1710, sampling volume 5 μl, measuring time 60 s, coefficient of variation = 3% (Pyne et al., 2000; Baldari et al., 2009; Tanner et al., 2010; Mamen, 2014)], after swimming was stopped for approximately 15 s to collect capillary blood samples from the hyperemirized earlobe (Finalgon®, Sanovi-Aventis Germany GmbH, Frankfurt am Main, Germany; Dassonville et al., 1998; Feliu et al., 1999). Afterward, the test was continued at the pre-rest speed until the blood sample was analyzed.

Figures 2A,B, 3 depict the algorithms for the TA, the FTA, and the LTD. After detection of the LT, swimming speed was slightly reduced (0.01–0.015 m/s) to stop lactate accumulation and to verify the MLSS_W_. Participants were instructed to rate exercise intensity on a scale from 1 to 10, 1 is defined as very light and 10 as the maximum load (Borg, 1982).

The test-protocol, data analyses, and documentation were executed automatically by custom written software. The digitized speed data were averaged across 5 s intervals and stored together with lactate levels, RPE, anthropometric, and time dependent data.

### Constant Speed-Test

The validity and reproducibility were assessed in a constant speed (CS)-test with 13 regional competitive swimmers/triathletes aged 19–32 years (26.2 ± 1.1 years, 5 females). Here, the initial speed was set 0.02 m/s below the previously determined swimming speed at the MLSS_W_ and increased by 0.01 m/s if the [La^−^] was constant (∆[La^−^] < 0.3 mM) in two consecutive 3-min steps. When a TC was realized, swimming speed was reduced by 0.01 m/s to stop or, at least, to stabilize the increase in [La^−^]. The test consisted of at least 10 steps with a break of 15 s in between. The RPE was recorded simultaneously.

### Validation of the Study Protocol in Swimming Competitions

**Figure 5:**
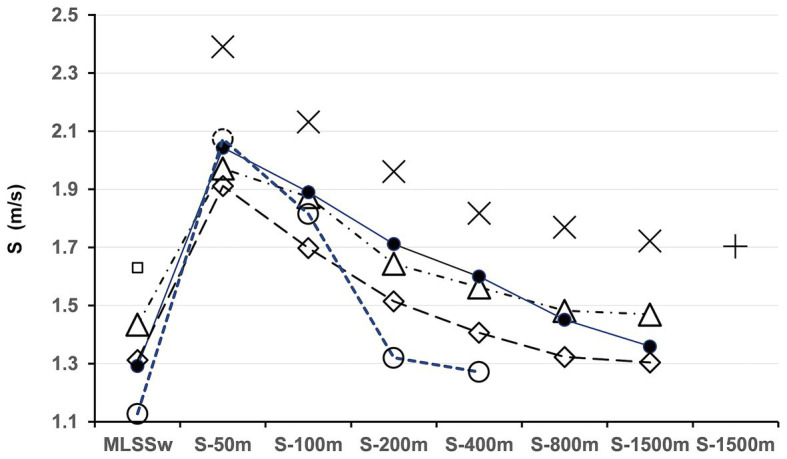
Comparison of swimming performance in relation to maximal lactate steady state workload (MLSS_W_) across Olympic distances. ▲ = Test person (TP) 1, ● = TP 2, ◊ = TP3, ○ = TP4, □ = estimated MLSS_W_ Australian National Team 1996, according to Pyne et al. (2001), + = world record 1994 (Kieren Perkins, Australia), X = current world records (FINA, F.I.d.N., 2021). TP1 had the highest MLSS_W_, resulting in comparatively good results across all distances. TP4 was slightly faster at 50 m, but slowed down rapidly at longer distances. Note that TP2 performed close to the MLSS_W_ in the 1,500 m race (∆ S-Comp/MLSS_W_ = −0.008 m/s). ∆S-Comp/MLSS_W_ for TP1/TP3 was moderate (0.035/0.067 m/s) with a relatively high MLSS_W_ for TP1 and a relatively low MLSS_W_ for TP3 (1.434/1.387 m/s; for a detailed analysis see section “Discussion”). Since MLSS_W_ data from world record holders were not available, we estimated the values using data from Pyne et al. (2001). Although these calculations are certainly not entirely accurate, they clearly demonstrate the large gap between MLSS_W1996_ and the 1,500 m world record in 1994. S, speed.

Seven to fourteen days before an official swimming competition (800 m women/1500 m men), all 22 subjects aged 13–23 years (17.3 ± 0.92 years, 13 women/9 men, swim training/strength training 8.5/2.4 h/week) conducted an ILT-test as described above. According to their own statements and based on their training and competition history, 10 athletes, aged 14–31 years (17.9 ± 1.6 years, nine females) were defined as long-distance swimmers. Twelve subjects, aged 13–26 (16.8 ± 1.1, six females) were assigned to the short-/middle-distance group. The group assignment based on the official FINA regulations (FINA, F.I.d.N., 2021). No information about the ILT-test results was given to the athletes before competition. The average swimming speed during competition was compared to ILT-test results. For the analysis of individual swimming performance, four male swimmers aged 17–22 years (19.8 ± 1 years) completed a full set of Olympic distances (50, 100, 200, 400, 1,500 m) and 800 m within 3–14 days after the ILT-test (Figure 5).

### Statistical Analysis

Statistical analyses were run in SPSS 24.0 (IBM). Data were analyzed by two-tailed paired and unpaired *t*-tests as appropriate. The F-test was used to test for equality of variances. Correlations between variables were assessed using Pearson’s correlation coefficient (*r*). Coefficient of variation (CV) was calculated for the ILT-/CS-test comparison. Data are shown as the mean ± SE unless otherwise stated. *p* < 0.05 were considered as statistically significant.

## Results

For running and cycling, we recently demonstrated that the equilibrium between lactate production and elimination and, thus, the MLSS_W_ can be disturbed by minute changes in exercise intensity (±0.1 m/s/7 W; Hering et al., 2018). Here, the athletes could implement such small changes in swimming speed by means of a visual light pacer mounted on the pool floor (Figure 1; see also section “Material and Methods”). In this way, we have determined LT and MLSS_W_ in 55 “individual lactate threshold-tests” (ILT-tests) in swimmers (Figures 4A,B).

We previously defined two threshold criteria (TC) for treadmill and bicycle ergometer tests based on an algorithm that has been developed into its current form by continuously incorporating ILT-test data (Hering et al., 2018). We applied the same procedure on swimming tests and, again, uncovered two scenarios of blood lactate accumulation at the LT (Figures 2A,B, 3; Hering et al., 2018). Either the arterial blood lactate concentration [La^−^] raised more than 0.5 mM in the exercise period directly after a WL increment (TC1) or it raised more than 0.4 mM 1–6 steps after the initial WL increment (TC2; Figures 2A,B, 3, 4A,B). Both, TC1 and TC2 were characterized by a steep increase in [La^−^], which occurred between absolute lactate levels of 1.8–7 mM (Figures 4A,B). The mean increase in [La^−^] was 1.1 ± 0.91 mM and the maximum increase was 5.1 mM (Figures 4A,B). In 4 out of 55 tests, the increase in [La^−^] did not reach 0.5 mM, but raised slowly in at least three successive steps at constant swimming speed (TC2a; Figure 3). After ILT detection, a slight workload reduction (0.01–0.015 m/s) stopped lactate accumulation and was used for MLSS_W_ verification (Figures 2A,B, 3, 4A,B). The rate of perceived exertion (RPE) raised or kept constant at high subjective fatigue levels after reaching the ILT (Figures 2A,B, 4A,B).

**Figure 6:**
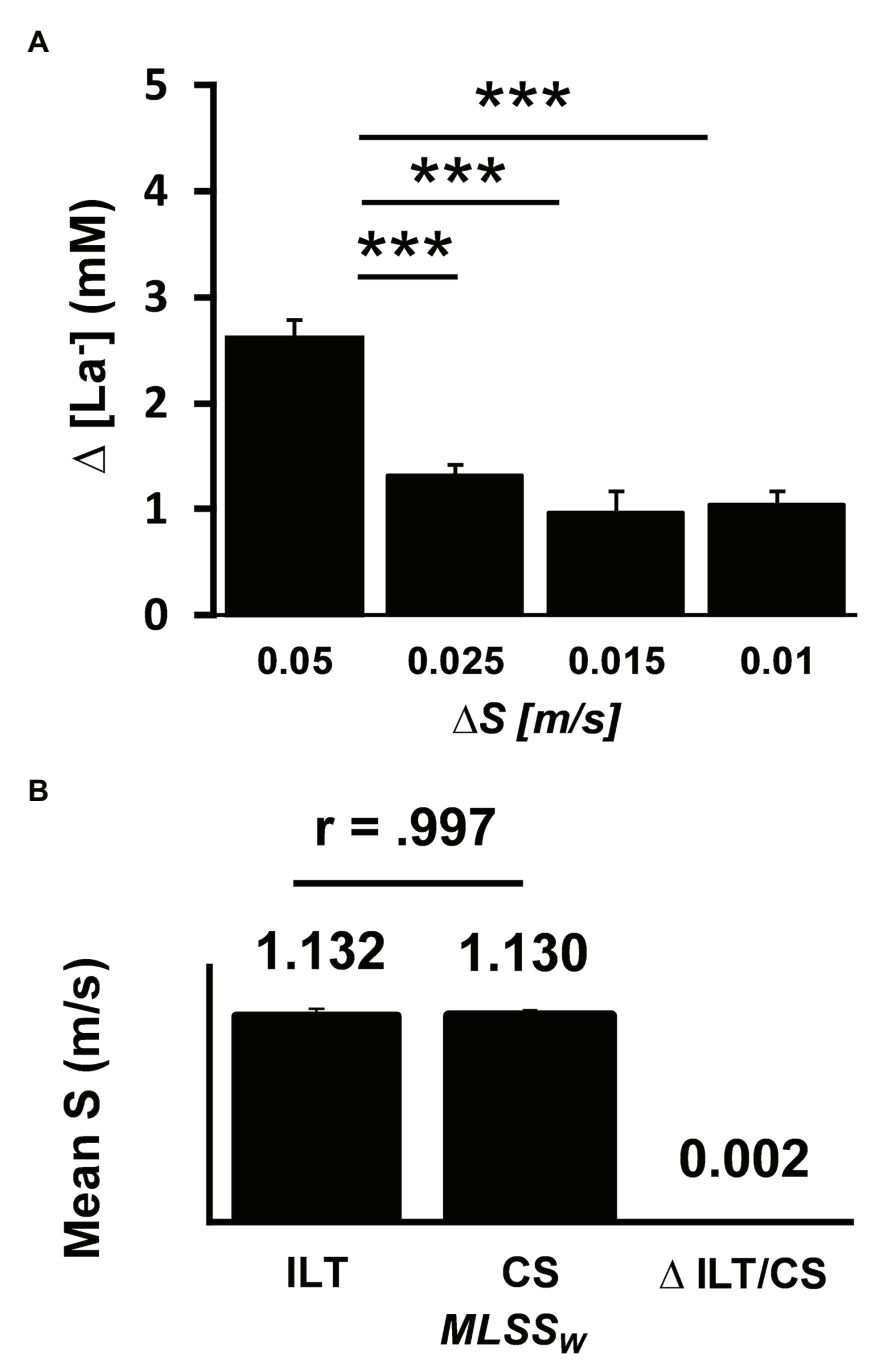
Lactate dynamics at the individual lactate threshold (ILT). **(A)** Relationship between workload (WL) variation (∆S) and blood lactate dynamics (∆[La^−^]) at the ILT using threshold criterion 1 and 2 (TC1/2). A rapid blood lactate accumulation (>0.4 Mm) occurs in response to a minute WL increase (0.01 m/s) after 1–6 steps at constant speed. While increments of 0.015 m/s and 0.025 m/s do not differ from 0.01 m/s, a step of 0.05 m/s provokes another significant lactate accumulation (0.05 m/s vs. 0.025 m/s; 0.05 m/s vs. 0.015 m/s, 0.05 m/s vs. 0.01 m/s, *N* = 65, ^***^p < 0.001, two-tailed unpaired *t*-tests). **(B)** Reliability and validity of the ILT-test. Intraindividual day to day comparison of the maximal lactate steady state workload (MLSS_W_) obtained by an ILT-test and a subsequent constant speed (CS)-test (ILT-test = 1.132 m/s, CS-test = 1.130 m/s, ∆ ILT-test/CS-test = 0.002 m/s, CV = 0.49%, *n* = 13, *r* = 0.997, *p* < 0.001). Data are mean ± SE. [La^−^], arterial lactate concentration; S, speed.

Notably, rapid blood lactate accumulation (>0.4 mM, TC1/2) occurred within six steps at constant pace, after swimming speed was increased very slightly (0.01 m/s). We next evaluated the impact of higher increments on lactate dynamics. While increments of 0.015 and 0.025 m/s do not differ from 0.01 m/s, a step of 0.05 m/s provoked another significant lactate accumulation (Figure 6A). These data show an extremely sensitive regulated lactate metabolism in swimming and indicate that minute changes in workload can rapidly unbalance lactate homeostasis.

To examine the validity and reproducibility of the ILT-test, we verified the results in a CS-test of at least 30 min near the MLSS_W_ (see section “Material and Methods”). Swimming speed at the MLSS correlated well between ILT-tests and CS-tests (Figure 6B). The minimal difference in average swimming speed at the MLSS between the two test protocols (0.002 m/s) was below the smallest workload increment used in the ILT-tests (0.01 m/s; Figure 6B). In addition, the test-retest coefficient (CC, 0.997) and the coefficient of variation (CV, 0.49%) are below the values known from the literature for day to day variability of the so-called “Gold Standard Method” (Hauser et al., 2013; CC = 0.98, CV = 3%; Batschelet, 2004; CV = 0.77%). This demonstrates the high reliability and validity of the ILT-test in accessing the MLSSw in swimming. As already shown for the ILT-test, increasing swimming speed by only 0.01 m/s resulted in a pronounced change in lactate kinetics (Figures 4C,D).

**Figure 7:**
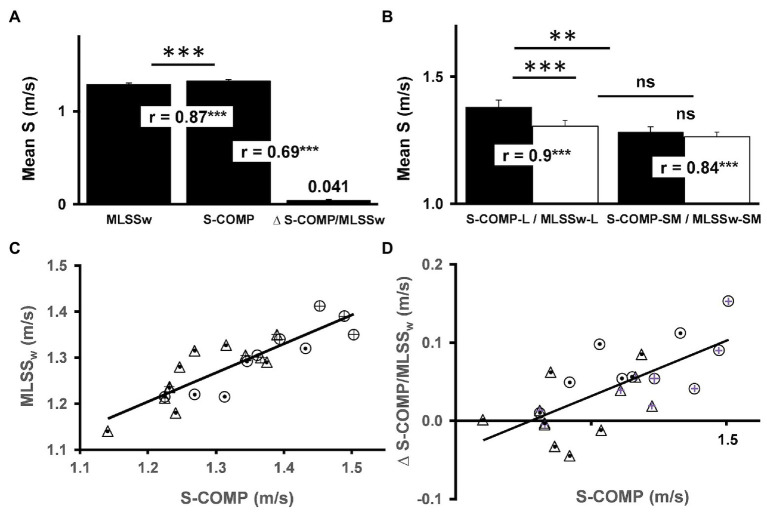
Comparison of test and race results. **(A)** Comparison of swimming speed at MLSS_W_ and swimming speed in competition (S-Comp) and their difference (∆ S-Comp/MLSS_W_; MLSS_W_: 1.283 m/s vs. S-Comp: 1.326, ^∗∗∗^*p* < 0.001, two-tailed paired *t*-test, *r* = 0.87, ^∗∗∗^*p* < 0.001; S-Comp: 1.326 m/s vs. ∆S-Comp/MLSS_W_: 0.041 m/s, *r* = 0.69, ^∗∗∗^*p* < 0.001; *n* = 22). **(B)** Comparison of mean swimming speed at MLSS_W_ and in competition (800 and 1,500 m, S-Comp) between long-distance swimmers (L, *n* = 10) and middle- and short-distance swimmers (SM, *n* = 12; S-Comp-L: 1.378 vs. MLSS_W_-L: 1.305, ^∗∗∗^*p* < 0.001, two-tailed paired *t*-test, *r* = 0.9, ^∗∗∗^*p* < 0.001; S-Comp-SM: 1.281 vs. MLSS_W_-SM: 1.264, ns., two-tailed paired *t*-test, *r* = 0.84, ^∗∗∗^*p* < 0.001; S-Comp-L: 1.378 m/s vs. S-Comp-SM: 1.281 m/s, ^∗∗^*p* < 0.01, two-tailed unpaired *t*-test; MLSS_W_-L: 1.305 m/s vs. MLSS_W_-SM: 1.264 m/s, ns., two-tailed unpaired *t*-test; ∆ S-Comp-L/MLSS_W_-L: 0.072 m/s vs. ∆ S-Comp-SM/MLSS_W_-SM: 0.015 m/s, ^∗∗^*p* < 0.01, two-tailed unpaired *t*-test). **(C)** Relationship between swimming speed at the MLSS_W_ and swimming speed in competition (*r* = 0.87, *p* < 0.001; ○ = long distance group (L), ∆ = middle and short distance group (SM), + = men, ● = women). **(D)** Relationship between ∆ S-Comp/MLSS_W_ and S-Comp (*r* = 0.698, *p* < 0.001). Data are mean ± SE. S, speed.

To evaluate the physiological relevance of the ILT-test in swimming, we compared the test results with data obtained from long distance competitions (800/1500 m). All swimmers performed the ILT-test, followed by a competition after at least 7 days of recovery. In accordance with our recent study on half-marathon runners (Hering et al., 2018), swimming speed in ILT-tests correlated with swimming speed in competition (Figures 7A,C). However, while the average running speed in a half-marathon was 5.2% below threshold speed (Hering et al., 2018), the swimmers performed 3.4% above their MLSS_W_ (S-Comp vs. MLSS_W_, Figures 7A,C). When the same analysis was applied separately for short-/middle-distance (S-Comp-SM, 50–400 m) and long-distance athletes (S-Comp-L, 800/1500 m), a ∆ S-Comp/MLSS_W_ only occurred in the long-distance group (S-Comp-L vs. MLSS_W_-L, Figure 7B). Finally, we found a close relationship between S-Comp and ∆ S-Comp/MLSS_W_ (∆ S-Comp/MLSS_W_ vs. S-Comp, Figure 7D), which is more pronounced in long-distance swimmers (∆ S-Comp-L/MLSS_W_-L vs. ∆ S-Comp-SM/MLSS_W_-SM, Figure 7B). This finding demonstrates the major role of supra-threshold capacity in swimming and very likely determines swimming performance on longer distances (S-Comp-L vs. S-Comp-SM, Figure 7B).

## Discussion

Along with biochemical and morphological adaptation, successful training stimuli integrate dimensions of time, movement quality, and exercise intensity to prime neuromuscular plasticity. Given the complexity of such highly individual and movement-specific processes, attention should be paid toward application of reliable, valid, and unbiased reference parameters, which capture exercise intensities in the form of speed (running and swimming) or power (cycling). There is compelling evidence that the MLSS_W_ is the most appropriate parameter to meet these requirements (Jamnick et al., 2020; for excellent reviews see also (Fluck and Hoppeler, 2003; Bishop et al., 2019). Although the MLSS_W_ has repeatedly been described as a reliable marker and accurate predictor of subthreshold endurance capacity in running and cycling (Heck et al., 1985; Hering et al., 2018; Jamnick et al., 2018), its relevance for supra-threshold intensities of exercise is unknown. Here, we have adapted a recently introduced single-visit test (ILT-test) for reliable determination of the MLSS_W_ in running and cycling for swimming and showed that swimming speed in competitions up to 1,500 m is well above the MLSS_W_. Moreover, absolute MLSS_W_ level and the difference between the MLSS_W_ and swimming speed in competition correlate well with general swimming performance, in particular with supra-threshold endurance capacity. Importantly, a training-related combination of a high MLSS_W_ and a high ∆ S-Comp/MLSS_W_ is more likely to be found in long-distance trained athletes than in short-distance trained athletes. Consequently, these data suggest that the MLSS_W_ is a reliable submaximal parameter for predicting endurance capacity below and above the LT and to set the optimal training intensity even in severe domains of exercise.

We recently provided a detailed discussion on physiological meanings of the ILT-test (Hering et al., 2018). Two issues are of particular interest and illustrate the relevance of the test procedure to exercise physiology: first, the extremely fine-tuned regulation of lactate homeostasis, which can be captured by the ILT-test. Minute workload variations while running, cycling, and swimming at the MLSS_W_ (0.1 m/s/7 W/0.01 m/s) generate drastic changes in blood lactate levels (Figure 6A). Second, ILT-test results are highly reproducible. The test-retest coefficients for running and swimming are 0.996 and 0.997, respectively (Hering et al., 2018).

We previously demonstrated a strong correlation between the MLSS_W_ and performance in half-marathon competition (*r* = 0.96; Hering et al., 2018). This allows a remarkably precise prediction of race results (Hering et al., 2018). Compared to half-marathon runners, we found a lower correlation between the MLSS_W_ and the average swimming pace in competition (S-Comp, 0.96 vs. 0.87). Hence, S-Comp in swimmers was on average 3.4% above the MLSS_W_ (data not shown), while half-marathon runners exercised 5.2% below (Hering et al., 2018). Moreover, we found differences in swimming speed (S-Comp-L vs. S-Comp-SM) and for the gap between MLSS_W_ and S-Comp (∆ S-Comp-L/MLSS_W_-L vs. ∆ S-Comp-SM/MLSS_W_-SM) when comparing long-distance (L, 400–1,500 m), and short-/middle-distance (50–200 m) athletes (SM; Figure 7B). The MLSS_W_ did not differ between the two groups (Figure 7B). Therefore, in contrast to half-marathon running, race results in swimming cannot be explained by the MLSS_W_ level alone. Rather, these data provide substantial evidence that the WL range above the MLSS_W_ is a major factor in swimming and probably in sports with comparable racing time. Thus, we speculate, that both, a high MLSS_W_ and a large ∆ S-Comp/MLSS_W_ are crucial in Olympic long-distance swimming (Figures 5, 7A,C,D). Finally, we found a stronger correlation (*r* = 0.9) between MLSS_W_-L and S-Comp-L compared to MLSS_W_-SM and S-Comp-SM (0.84; Figure 7B), while only the correlation between ∆ MLSS_W_-L/S-Comp-L vs. S-Comp-L was significant (*p* < 0.05; data not shown). In long-distance swimmers, this indicates an increased fatigue resistance of the muscle fibers that are recruited above the MLSS_W_.

With increasing WL demands at the MLSS_W_, fast-twitching and more glycolytic type II fibers get more likely recruited (Henneman et al., 1965; Burke et al., 1971; Peter et al., 1972; Vollestad and Blom, 1985). The accompanying blood lactate accumulation will rapidly exceed clearance systems and is commonly referred to as LT. Exercise in this intensity range can trigger the following adaptions: (i) higher mitochondrial content and/or activity (Dudley et al., 1982; Howald et al., 1985; Hoppeler, 1986; Daussin et al., 2008), increased capillarization (Li et al., 2020), (ii) higher MCT1/MCT4 content (Dubouchaud et al., 2000; de Araujo et al., 2015) and (iii) an overall increase in lactate clearance rate (Bergman et al., 1999; Messonnier et al., 2013; for comprehensive reviews, see Fluck and Hoppeler, 2003; Schiaffino and Reggiani, 2011; Hoppeler, 2016). These adaptions may at least partially explain the higher fatigue resistance above the MLSS_W_ of long-distance swimmers (800–1,500 m).

Fast-twitching muscle fibers consume considerably more ATP and O_2_ (Greenhaff et al., 1993; Stienen et al., 1995; He et al., 2000; Szentesi et al., 2001). Converting them into muscles with a higher aerobic capacity to expand supra-threshold endurance capacity is challenging in humans, since everyday movements counteract eventual training interventions (Monster et al., 1978; Lindstedt et al., 1991; Pette and Staron, 1997). A conversion of muscle fibers from MHC IIa type to MHC I type, with the same or larger fiber diameter has been demonstrated in cyclists (Coyle et al., 1992; Horowitz et al., 1994; Coyle, 2005) and in a bicycle ergometer training-study at 75% peak oxygen consumption (Dubouchaud et al., 2000). Importantly, the plasticity of muscles was correlated with higher endurance capacity (Coyle et al., 1992; Horowitz et al., 1994; Coyle, 2005) possibly because the ATP consumption of type I fibers is comparatively lower for the same force production (Greenhaff et al., 1993; Stienen et al., 1996; He et al., 2000; Szentesi et al., 2001). However, there would only be a net advantage if the discipline-specific movement execution corresponds to the twitching properties of performance-relevant muscles/muscle fibers (Tesch and Karlsson, 1985). To our knowledge, no bioptically proven, training-induced fiber transformation in swimming has been reported so far. Comparison of highly-trained elite 1,500 m swimmers and amateurs demonstrated larger and more type I fibers in the M. vastus lateralis of elite swimmers (Gerard et al., 1986). Although exercise induced fiber transformation from fast to slow after several years of training is a possible explanation for the higher S-Comp of long-distance swimmers, data on arm muscles is not available.

Recent studies show that training stimuli in the MLSS_W_ range (Dubouchaud et al., 2000) induce not only muscle fiber transformation but also prime neuronal plasticity in severe domains of exercise (Pin-Barre et al., 2017; Rahmati and Kazemi, 2019; Constans et al., 2021). Complicating this picture, metabolic homeostasis in swimming is more dependent on technical skills than in running or cycling (Figueiredo et al., 2014). For example, elite swimmers use different combinations of stroke parameters compared to amateur-level athletes (Seifert et al., 2010b). Moreover, several studies consistently found increased stroke frequency accompanied by decreased stroke length slightly above the MLSS_W_ (Oliveira et al., 2012; Figueiredo et al., 2013, 2014; Pelarigo et al., 2017). Alberty et al. assume that a change in arm coordination has two key benefits: (i) a better chain of the propulsive actions and (ii) a greater time allotted to propulsion per distance unit during increasing muscular fatigue (Alberty et al., 2009; Seifert et al., 2010a).

A study on open water swimmers (OWSs, 5–25 km) and short distance swimmers (SDSs, 50–100 m), two subgroups comparable to the present study, clearly demonstrates the strong relationship between muscle coordination and energy metabolism (Seifert et al., 2010a). In an intensity-adjusted progressive 6 × 300 m test, the OWS achieved a higher mean speed (1.39 vs. 1.27 m·s^−1^), stroke rate (0.62 vs. 0.55 Hz), and stroke index (SI = SL·SV, 3.19 vs. 2.96 m^2^ s^−1^), and a lower stroke length (SL, 2.28 vs. 2.31 m·stroke^−1^) and index of coordination (IdC, −21.7 vs. −11.2%) than the SDS. When a lag time occurred between the propulsive phases of the two arms, the stroke coordination was called as “catch-up” (IdC < 0%; Chollet et al., 2000). Importantly, the OWS, who should be highly fatigue resistant, kept their coordination in great catch-up, presumably because they were highly focused on their hydro-dynamic position in order to minimize resistive forces and maximize efficiency (Seifert et al., 2010a). The relationship between increasing energy consumption and decreasing catch-up phase was observed in both groups [IdC vs. energy cost (C), *p* < 0.05], but the OWS had longer glide phases in catch-up mode (IdC) and higher stroke efficiency (SI) for the same energy consumption, resulting in more efficient propulsion. Moreover, on the average of six progressive 300 m sets beginning with a swimming speed near the MLSS_W_ (0.1 m/s below the 400 m best time, increment = 0.017 m/s), the OWS also had lower blood lactate concentrations (3.1 vs. 5.9 mmol·L^−1^), lower energy consumption (C, 12.89 vs. 14.8 J·kg^−1^·m^−1^), and a greater net VO_2_ (2,903 vs. 2,825 ml·min^−1^) than the SDS (Seifert et al., 2010a). This is in line with our data showing a higher S-Comp and a larger ∆ S-Comp/MLSS_W_ in 800–1,500 m swimmers. In accordance with Seifert et al. (2010a), we hypothesize that long-distance trained swimmers, and likely endurance athletes with comparable racing times, have a higher fatigue resistance in the supra-threshold range due to improvements in technique and metabolism. Training intensity/duration adjustment in moderate to severe domains of exercise will trigger neuromuscular adaptations (Dudley et al., 1982; Dubouchaud et al., 2000; Messonnier et al., 2013; Figueiredo et al., 2014; de Araujo et al., 2015), which most likely manifest in a higher MLSS_W_ (Billat et al., 2004; Philp et al., 2008). Such a higher fatigue resistance in the supra-threshold range can facilitate economic movement over longer periods of time.

Together with the improved coordination patterns described above, peripheral and/or central recruitment of additional motor units and their higher discharge frequencies can be converted into strength increases (Henneman et al., 1965; Sale, 1988; Wakeling et al., 2002, 2006, 2012; Jensen et al., 2005; Ansdell et al., 2020). This ultimately leads to higher muscle power (Power = Force · Velocity/Speed) with a constant fiber/muscle cross-section and thus also a constant weight (Gabriel et al., 2006). A further increase in strength can be achieved by increasing the cross-sectional area of muscles through unspecific or specific strength training (Fyfe et al., 2014). However, it is important to note, that the isolated stimulation of muscle hypertrophy neglects neuromuscular optimization, which limits its application in competitive sports. Nevertheless, an elevated fiber/muscle cross-section will likely result in improved performance if ∆MLSS_W_/S-Comp increases at low or constant MLSS_W_. Such a scenario may also explain the performance profile of TP4 (Figure 5, see also the next paragraph).

An in-depth analysis of complete sets of Olympic distances (Figure 5) reveals significant differences in MLSS_W_ scores and for the gap between MLSS_W_ and swimming pace in competition (∆ S-Comp/MLSS_W_). The comparatively high MLSS_W_ of TP1 indicates substantial neuromuscular adaptations, which may explain the race results above the average in all disciplines. Other athletes (TP2-4) seem to have less well coordinated movement patterns, likely underlying their lower threshold speeds (MLSS_W_). TP4 has the fastest 50 m time, but a considerably lower MLSS_W_ manifesting in a rapid decline in performance starting from 200 m. Although at a lower level, ∆ S-Comp/MLSS_W_ of TP2 follows the course of TP1 indicating a similar history of training stimuli. In contrast, the relatively high ∆ S-Comp/MLSS_W_ for distances up to 400 m and the decline in speed at 800 and 1,500 m of TP4 suggests insufficient aerobic neuromuscular adaptation of muscle fibers involved in supra-threshold exercise.

## Conclusion

Considerable progress has been made in understanding the molecular and neuromuscular architecture of adaptation processes to various training stimuli (Fluck and Hoppeler, 2003; Fyfe et al., 2014; Granata et al., 2018; Bishop et al., 2019). However, successful translation of basic insights into useful training concepts has been constrained by the lack of appropriate reference parameters (Jamnick et al., 2020). Using a sophisticated single-exercise test in swimming and results from swimming competitions, we provide clear evidence that the MLSS_W_ is a reliable and valid parameter to assess endurance capacity in moderate to severe domains of exercise (Hering et al., 2018; Jamnick et al., 2020). Consequently, we suggest that personal training recommendations and monitoring of exercise-induced changes in individual performance level should be calculated relative to the MLSS_W_ rather than to peak or maximal workload parameters (Baldwin et al., 2000; Skorski et al., 2012; Bishop et al., 2019).

## Data Availability Statement

The raw data supporting the conclusions of this article will be made available by the authors, without undue reservation.

## Ethics Statement

The studies involving human participants were reviewed and approved by the Ethics Committee (institutional review board, IRB) of the University of Konstanz. Written informed consent to participate in this study was provided by the participants’ legal guardian/next of kin.

## Author Contributions

GH developed the hardware and software, conceived and designed research, performed experiments, analyzed data, prepared and interpreted results, drafted the manuscript, prepared figures, and edited and revised the manuscript. JS performed experiments, interpreted results, prepared figures, and edited and revised the manuscript.

### Conflict of Interest

The authors declare that the research was conducted in the absence of any commercial or financial relationships that could be construed as a potential conflict of interest.
